# PReS-FINAL-2203: Assessment of sleep problems in children with familial Mediterranean fever

**DOI:** 10.1186/1546-0096-11-S2-P193

**Published:** 2013-12-05

**Authors:** B Makay, SK Kılıçarslan, A Anık, E Ünsal

**Affiliations:** 1 Dokuz Eylül University Hospital, Izmir, Turkey

## Objectives

The study aimed to investigate sleep patterns, sleep disturbances and possible factors that are associated with sleep disturbances among children with FMF.

## Methods

Forty six FMF patients and 80 age- and sex-matched healthy children were enrolled in the study (Table 1). The patients who had an attack during the last 2 weeks were not included. Demographic data, FMF symptoms, disease duration, dose of colchicine, disease severity score, number of attacks in the last year, MEFV mutation and serum C-reactive protein level were recorded for each patient. *Children's Sleep Habits Questionnaire *was performed. It is a parent-report questionnaire assessing the typical sleep patterns of children. It includes 33 items measuring sleep disturbances (8 subscales) and 3 three items collecting information about bedtime, wake-up time and sleep duration over a "typical" recent week. A total score of ≥41 defines "clinically significant sleep disturbance".

**Table 1:** 

	FMF patients	Healthy controls	p value
Length of wakings (minute)	9.5 ± 18.6	3.1 ± 3.6	**0.026**

Total sleep duration (hour)	9 ± 1.4	8.8 ± 1	0.34

Total sleep score *SUBSCALES*	50.1 ± 10.4	46.6 ± 6.6	**0.010**

Bedtime resistance Sleep-onset delay Sleep duration Sleep anxiety Nightwakings Parasomnias Sleep-disordered breathing Day-time sleepiness	8.6 ± 3.8 2.4 ± 2.1 3.8 ± 1.3 6.1 ± 2.4 4.7 ± 1.6 8.9 ± 2.1 4 ± 1.7 14.1 ± 4.9	7.8 ± 2.2 1.4 ± 0.7 4 ± 1.3 5.4 ± 1.7 4 ± 1 8.9 ± 1.8 3.4 ± 0.8 14.7 ± 3.7	0.114 **0.014** 0.463 0.054 **0.002** 0.991 **0.006** 0.441

## Results

The total sleep scores of the patients with FMF were significantly higher than the control group. Total sleep duration were similar between 2 groups. The comparison of subscale scores were given in table. Gender and age had no effect on total sleep scores in both groups. There was not a significant corelation between the total sleep score and disease duration, dose of colchicine, disease severity score, number of attacks in the last year, and serum C-reactive protein level in FMF patients. Besides, the patients with exercise-induced myalgia (n = 21) had significantly higher sleep scores than the patients without (n = 25) (54.8 ± 11.3 vs 46.3 ± 7.8, p = 0.008).

## Conclusion

This is the first study investigating sleep patterns, sleep disturbances and possible factors that are associated with sleep disturbances among children with FMF. The results of this study suggested that exercise-induced myalgia might contribute to sleep disturbances in FMF as well as ongoing subclinical inflammation.

## Disclosure of interest

None declared.

